# Brevilin A Inhibits VEGF-Induced Angiogenesis through ROS-Dependent Mitochondrial Dysfunction

**DOI:** 10.1155/2022/5888636

**Published:** 2022-12-14

**Authors:** Bailing Wei, Zhen Hao, Hao Zheng, Yuanhua Qin, Feng Zhao, Lei Shi

**Affiliations:** College of Basic Medical Sciences, Dalian Medical University, No. 9 West Section Lvshun South Road, Dalian 116044, China

## Abstract

Brevilin A (BA), a sesquiterpene lactone isolated from *Centipeda minima* herb, has been identified to exhibit potent anticancer activity. However, the potential pharmacological effect and mechanism of BA in regulating endothelial cell (EC) angiogenesis, a key event in tumor growth, is poorly understood. In this study, BA was shown to significantly prevent vascular endothelial growth factor (VEGF) induced EC angiogenic capacities *in vitro*, *ex vivo,* and *in vivo*. Subsequent functional assays revealed that BA dose dependently inhibited VEGF-stimulated survival, proliferation, migration, and triggered apoptosis activity in human umbilical vein endothelial cells (HUVECs), as well as suppressed the expression of antiapoptotic protein Bcl-2, increased the expression of proapoptotic protein caspase-3 and Bax, and suppressed PI3K/AKT pathway. Meanwhile, BA was also able to depolarize mitochondrial membranal permeability (MMP), accelerate mitochondrial superoxide accumulation, induce intracellular reactive oxygen species (ROS) production, and decreased intracellular glutathione (GSH) in HUVECs. Furthermore, both mitochondria-specific superoxide scavenger Mito-TEMPOL and broad-spectrum antioxidant N-acetyl-cysteine (NAC) dramatically abolished BA-induced mitochondrial dysfunction and mitochondrial ROS production, causing the reversion of PI3K/AKT pathway and repression of apoptosis, eventually correcting the impaired endothelial behavior in survival, growth, migration, and angiogenesis. Collectively, our data for the first time identified a new mechanism for antiangiogenic effect of BA in vascular EC, one that is based on the regulation of mitochondrial-dependent ROS overproduction.

## 1. Introduction

Plant-derived natural products have been well-recognized to display powerful therapeutic effects on various human diseases [1]. Among them, the antitumor activity of natural products has received increasing attention at present [2]. Brevilin A (BA), a natural sesquiterpene lactone, is purified from *Centipeda minima* herb, a Traditional Chinese Medicine (TCM) initially used to relieve stuffy nose, asthma, and cough [3]. Recent studies have revealed the great efficacy of BA on antitumor activities, particularly those of the breast cancer [3, 4], gastric cancer [5], colon carcinoma [6], lung cancer [7, 8], glioblastoma [9], melanoma [10], multiple myeloma [11], and nasopharyngeal carcinoma [12]. Specifically, BA inhibits proliferation, migration, and invasion, in part, through suppressing signaling pathways, including JAK2/STAT3 and PI3K/AKT/mTOR [3, 4, 6, 7, 13]. Moreover, BA also induces mitochondrial apoptosis via increasing NOX2 and NOX3-mediated reactive oxygen species (ROS) generation [3, 7].

Angiogenesis (blood vessel sprouting), the sprouting of new capillaries from endothelial cells (EC) in preexisting vessels, plays a crucial role in embryonic vascular development and differentiation, wound healing, and organ regeneration [14, 15]. It also contributes to the progression of tumor growth and metastasis that depend on neovascularization, which is initiated by chemical signals from carcinoma cells in a rapidly growing phase [16]. In reality, this signaling activates certain genes in surrounding the normal host tissue that causes proteins, such as proangiogenic factors, to encourage the growth of new blood vessels of solid tumors [17]. Vascular endothelial growth factor (VEGF), a proangiogenic factor, is a key mediator for the proper tumor vessel development, in which it is upregulated by oncogene expression, growth factors, and hypoxia [18–20]. VEGF mediates vascular EC survival, permeability, proliferation, and migration by binding and activating autophosphorylation of the tyrosine kinase receptors (VEGFR-2) [21–23]. Owing to this fact, tumor-associated angiogenesis can be mimicked by inducing human umbilical vein endothelial cells (HUVECs) with VEGF [24]. A series of antiangiogenic drugs have been invented and approved for clinical applications against different cancers, such as antibodies (bevacizumab, ranibizumab, and ramucirumab), small kinase inhibitors (lenvatinib, pazopanib, and sorafenib), and fusion proteins (aflibercept) [25–28]. However, clinical observations have shown that these reagents may cause a broad spectrum of toxic side effects and unsatisfied pharmacokinetic behavior, and eventually develop resistance and limit therapeutic outcomes [29, 30]. Therefore, novel medicines with higher efficacy and minor toxic side effects against tumor angiogenesis are urgently warranted.

Given the fact that natural products, especially from plants, are valuable resources for seeking leading compounds against human diseases; we noticed that numerous studies have indicated the important roles of natural products in modulating tumor angiogenesis [24, 31, 32]. Numerous preclinical studies demonstrated BA being of great advantages against xenograft tumor growth in animal models, and its pharmacological actions are largely detected in cancer cells, while, it remains unknown whether BA can inhibit tumor-associated angiogenesis, a key process and hallmark in solid neoplasm progression [33]. Therefore, the current study examined the potential effects of BA on *in vitro*, *ex vivo,* and *in vivo* endothelial angiogenesis and uncovered the possible mechanism underneath.

## 2. Materials and Methods

### 2.1. Antibodies and Reagents

Rabbit anti-NOX2 (Cat No: AF7596) and anti-NOX4 (Cat No: AF1498) antibodies were purchased from Beyotime Biotechnology. Rabbit anti-p-PI3K (Cat No: 4228S) and anti-PI3K (Cat No: 4292) antibodies were purchased from Cell Signaling Technology. Rabbit anticleaved caspase-3 (Cat No: 19677-1-AP), anti-p-AKT (Cat No: 28731-1-AP) and anti-AKT (Cat No: 60203-2-Ig) antibodies, and mouse anti-Bax (Cat No: 60267-1-Ig) monoclonal antibodies were purchased from Proteintech. Rabbit anti-Bcl-2 (Cat No: CAS7511) antibody was purchased from Bioworld. Mouse anti-*β*-actin (Cat No: A01010) antibody was obtained from Abbkine. Mouse anti-BrdU (Cat No: 66241-1-Ig) monoclonal antibody was purchased from BD Biosciences, and FITC-Lectin (Cat No: L9381) was purchased from Sigma.

Brevilin A (Cat No: CFN99694) with the highest purity grade of >98% was purchased from ChemFaces. FBS (Cat No: 10099141C) was obtained from Gibco (Thermo Fisher). Trypsin (Cat No: C0201) with EDTA was obtained from Beyotime Biotechnology. Penicillin and streptomycin (Cat No: P1400) and dimethyl sulfoxide (DMSO) (Cat No: D8371) were purchased from Solarbio Biotechnology. MTT (Cat No: CM7461) was purchased from Coolabor, and protease inhibitor cocktail (Cat No: P8465) was obtained from Sigma. Mitochondrial membrane potential assay kit (JC-1) (Cat No: M8650) was purchased from Solarbio Biotechnology. H2DCFDA (Cat No: C400) and MitoSOX (Cat No: M36008) were purchased from Invitrogen. Annexin V-FITC apoptosis detection kit (Cat No: E-CK-A211), crystal violet stain, and N-Acetyl-L-cysteine (NAC) (Cat No: ST2524) were purchased from Beyotime Biotechnology. Mito-TEMPOL (Cat No: MX4808) was purchased from Maokang Biotechnology. VEGF (Cat No: 100-20) was obtained from PeproTech.

### 2.2. Animal and Ethical Statements

C57BL/6 male mice (8-10 weeks old) were used in this study. Animals were maintained at Dalian Medical University Laboratory Animal Center under the specific pathogen-free condition, and all animal studies were approved by the Ethics Committee for Biology and Medical Science of Dalian Medical University.

### 2.3. Matrigel Plug Assay

Matrigel plug assay was modified from our previous report [34]. Briefly, we injected 0.5 mL Matrigel containing 100 ng murine VEGF and 20 units of heparin with BA (100 *μ*M) or solvent (0.1% DMSO) into the dorsal subcutaneous area of mice. Matrigel plugs were removed 7 days after implantation. Frozen sections from the plugs (10 *μ*m) were prepared for determining neovascularization, and vascular densities were visualized by FITC-Lectin positive area.

### 2.4. Cell Culture

Primary human umbilical vein endothelial cells (HUVECs) were isolated as previously described [34, 35]. Briefly, HUVECs were cultured in ECM medium supplemented with 5% FBS, 1% endothelial cell growth supplement (ECGS), and 1% penicillin/streptomycin and grown at 37°C in a humidified 5% CO_2_ atmosphere.

### 2.5. Tube Formation Assay

Tube formation assay was performed as described in our previous study [35]. After BA preincubation at the indicated concentrations, HUVECs suspensions (50 *μ*L, 4 × 10^5^/mL) in 0.1% BSA/ECM containing VEGF (30 ng/mL) were seeded on ibiTreat angiogenesis slides (Ibidi) precoated with 10 *μ*L Matrigel (REF354248, Corning), and the formation of tubular like structure was observed by an inverted microscope at 6 h. The angiogenic capacities were evaluated by accumulative lengths of total tubes, the area occupied by tubes, and the number of branching points.

### 2.6. Spheroid Sprouting Assay

A spheroid sprouting assay was performed as per our previous publication [34]. Cell suspension (1.6 × 10^3^/mL) was prepared in ECM medium containing 5% FBS and 0.24% high viscosity carboxymethyl cellulose (9004-32-4, Sigma,). Each spheroid was generated by hung-drop cultures of 25 *μ*L cell suspension for 24 h. The spheroids were collected with PBS containing 5% FBS and harvested by a 5 min centrifugation at 1000 rpm. The spheroids were further embedded into collagen working solution containing rat tail collagen I (2 mg/ml, 354236, Corning) buffered with 10× medium 199 (M650, Sigma), and 0.5 M NaOH was used to adjust pH to 7.4, which was allowed to polymerize at 37°C for 30 min. An equal volume of ECM with 5% FBS and indicated compounds (as two folds as final concentration) were added to the polymerized collagen gels. Endothelial spheroid sprouting was visualized by an inverted microscope 24 h later. The angiogenic abilities were quantified by accumulated sprout lengths from each endothelial spheroid.

### 2.7. Aortic Ring Assay

An aortic ring assay was performed as per our previous report [34]. Aortic rings (1 mm in width) were prepared from C57BL/6 male mice and embedded in 25 *μ*L collagen working solution as described in spheroid sprouting assay. After polymerization at 37°C for 30 min, ECM with 2.5% mouse serum and murine VEGF (30 ng/mL) was applied for *ex vivo* aortic ring cultures. BA alone or combinations with other reagents was included in the culture medium. After 7 days of cultures at 37°C in a humidified 5% CO_2_ atmosphere, endothelial sprouts from rings were visualized by using FITC-Lectin (L2895, Sigma) and further evaluated by the length of the accumulated sprouts.

### 2.8. MTT and BrdU Incorporation Assays

HUVECs were seeded into 96-well plates (5000 cells/well) with BA in the indicated concentrations. For some experiments, Mito-TEMPOL (2 *μ*M) and NAC (3 mM) were preincubated 30 min before BA treatment. MTT assays were performed as described in our previous study [35]. Briefly, the reagent with a final concentration of 0.5 mg/mL was applied into cell cultures for 2 hours, followed by replacement of culture medium with 100 *μ*L DMSO, and the O.D. values were determined at 490 nm by using the Thermo Scientific Varioskan LUX.

For BrdU incorporation assay, HUVECs were incubated with BrdU (30 *μ*M, Cat. 19-160, Sigma) for 2 h in the dark. Following fixation with 4% PFA and permeabilization with 0.1% BSA/0.5% Triton X-100/PBS, primary antibody against BrdU (66241-1-Ig, Proteintech) and Alex594-IgG secondary antibody (8890, Cell Signaling) were sequentially applied to visualize BrdU incorporation under a fluorescence microscope (BX53, Olympus) and evaluated by percentages of BrdU-positive cell population.

### 2.9. Cell Migration Assay

Cell treatment with BA or combination with Mito-TEMPO and NAC were identical with proliferation assay. Scratch wound-healing and Transwell migration assays were conducted as previously described [35, 36]. For scratch wound-healing assay, HUVECs monolayers were scratched using a sterile 200 *μ*L pipette, and cell migration images were separately recorded the same field at initial and 12 hours later. The net migrating area within 12 hours was calculated by using Image J.

For Transwell migration assay, HUVECs suspension in 5% FBS ECM (500 *μ*L, 10000/mL) was seeded into the upper chamber with 8 *μ*m-pore size (Corning, REF363097) to form full monolayer and maintained in 37°C with humidified 5% CO_2_ atmosphere for 12 hours following the presence of 30 ng/mL VEGF/5% FBS ECM in the lower chamber. The upper layer cells were removed from the Transwell chamber, washed twice with PBS, fixed in 4% PFA for 15 min, then stained with 0.1% crystal violet solution and microscopically observed for the number of migrating cells.

### 2.10. Apoptosis Assay

Apoptosis was measured by Annexin V-FITC apoptosis assay kit (E-CK-A211, Elabscience) according to manufacturer's instructions. Briefly, HUVECs were seeded at 2 × 10^5^ cells per well in a 6-well plate and treated with indicated concentrations of BA in the presence or absence of Mito-TEMPOL (2 *μ*M) and NAC (3 mM) in 6-well plates for 12 h. Cells were stained with FITC-labeled Annexin V and PI at room temperature for 20 min in the dark and analyzed using the BD Accuri C6 Plus flow cytometry.

### 2.11. Western Blot

Western blot was conducted as previously described [37]. Briefly, HUVECs were subjected to SDS-PAGE and transferred to nitrocellulose membranes. After incubation with specific primary antibodies and secondary antibodies of antimouse Dylight 680 (C90219-05, LI-COR) or antirabbit Dylight 800 (90220, LI-COR), the membranes were then scanned using the Odyssey CLx Imaging System (LI-COR), and the images were generated employing the Image Studio software.

### 2.12. Measurement of Mitochondrial Membrane Potential (MMP)

MMP was measured by mitochondrial membrane potential assay kit with JC-1 (M8650, Solarbio) according to manufacturer's instructions. Briefly, HUVECs were treated with indicated concentrations of BA in the presence or absence of Mito-TEMPOL (2 *μ*M) and NAC (3 mM) in 6-well plates for 12 h. After that, the cells were harvested, washed with PBS, and incubated with the JC-1 fluorescent probe for 20 min in the dark at 37°C. Following incubation, the cells fluorescence intensity was detected using the BD Accuri C6 Plus flow cytometry.

### 2.13. Measurement of Mitochondrial Superoxide Generation

Mitochondrial superoxide contents were measured using the MitoSOX kit (Invitrogen) according to the manufacturer's instructions. Briefly, HUVECs were treated with indicated concentrations of BA in the presence or absence of Mito-TEMPOL (2 *μ*M) and NAC (3 mM) for 12 h. After that, the cells were harvested and incubated with 5 *μ*M MitoSOX reagent to incubate cells for 10 min in the dark at 37°C. The cell's mean fluorescence intensity (MFI) was then detected using the BD Accuri C6 Plus flow cytometry.

### 2.14. Measurement of Intracellular Reactive Oxygen Species (ROS) Generation

Intracellular ROS generation was measured by a 2,7-dichlorodihydrofluorescein diacetate (H2DCFDA) kit (Invitrogen) according to the manufacturer's instructions. Briefly, HUVECs were seeded and treated with indicated concentrations of BA in the presence or absence of Mito-TEMPOL (2 *μ*M) and NAC (3 mM) in 6-well plates for 12 h. After that, the cells were washed with PBS and incubated with H2DCFDA for 15 min in the dark. Following incubation, the cell's MFI was detected using the BD Accuri C6 Plus flow cytometry.

### 2.15. Measurement of Intracellular Glutathione (GSH)

Intracellular GSH was measured by a microreduced glutathione assay kit (Solarbio) according to the manufacturer's instructions. Briefly, HUVECs were seeded and treated with indicated concentrations of BA in the presence or absence of Mito-TEMPOL (2 *μ*M) and NAC (3 mM) in 6-well plates for 12 h. After drug treatment, the cells were harvested and detected at 405 nm using the Thermo Scientific Varioskan LUX. The GSH contents were expressed as *μ*g/mg prot.

### 2.16. Statistical Analysis

Data are presented as means ± SEM of at least three independent experiments. Statistical analysis was performed using GraphPad Prism 8 software with one-way ANOVA followed by Bonferroni post-hoc test. The values of *p* < 0.05 or less were considered statistically significant.

## 3. Results

### 3.1. Brevilin A Inhibits Endothelial Angiogenesis

**Figure 1:**
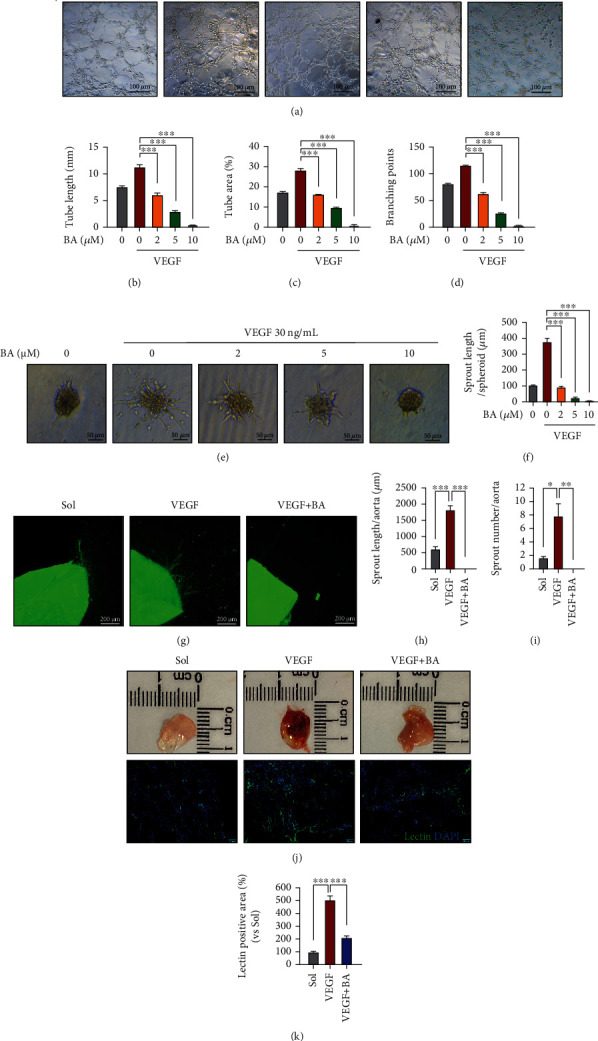
The effects of Brevilin A on angiogenesis. (a–d) The representative images showing the capacities of BA (0-10 *μ*M) on tube formation of HUVECs in the presence of VEGF (30 ng/mL), and the accumulated endothelial tube length, tube area, and branching points were quantified (scale bars: 100 *μ*m; *n* = 5). (e, f) The representative images showing the capacities of BA (0-10 *μ*M) on spheroid sprouting of HUVECs in the presence of VEGF (30 ng/mL), and the accumulated endothelial tube length was quantified (scale bars: 50 *μ*m; *n* = 8). (g–i) The effects of BA (10 *μ*M) in endothelial sprouting from isolated aortic rings cultured in modified collagen matrix with stimulation with VEGF (30 ng/mL), and endothelial sprouts were visualized by isolectin B4 staining (Green) (scale bars: 200 *μ*m; *n* = 3). (j, k) The appearance of VEGF (200 ng/mL) containing Matrigel plugs impregnated with BA (100 *μ*M) or solvent (0.1% DMSO) was isolated in 7 days after implantation into the dorsal subcutaneously area of mice (*n* = 3 mice per group), and vascular densities of plugs were assessed by lection-positive cells from their frozen sections (green: BrdU; blue: DAPI. Scale bars: 50 *μ*m). The graphs summarize the data from at least three times independent experiments. Data are shown as the mean ± SEM. One-way ANOVA with Bonferroni post-hoc test and ^∗^*p* < 0.05, ^∗∗^*p* < 0.01, and ^∗∗∗^*p* < 0.001 versus solvent (Sol).

To examine the impact of BA (chemical structure shown in Figure S1) on endothelial angiogenesis, primary HUVECs were firstly treated with BA as indicated doses (0-10 *μ*M) and further subjected to *in vitro* angiogenic assays. We found that BA would dose dependently prevent HUVECs from VEGF-stimulated tube-forming capacities on the Matrigel surface (Figures 1(a)–1(d)). Besides, although VEGF promoted outgrowth of human endothelial spheroids embedded in collagen matrix, the effect was greatly disrupted in BA-treated cells (Figures 1(e) and 1(f)). Moreover, angiogenic abilities of native EC were determined by an aortic ring sprouting assay, in which VEGF increased endothelial sprouting from isolated aortic rings in number and accumulated length (lectin B4^+^ for EC); however, the response was greatly reduced by BA treatment (Figures 1(g)–1(i)). To further confirm our results *in vivo*, we evaluated the neovascularization in Matrigel plugs containing either solvent or BA, 7 days after subcutaneous implantation into mice, and found that plugs impregnated with BA exhibited far less isolectin B4-positive cells over control plugs (Figures 1(j) and 1(k)), indicating impaired vascularization. Herein, our data demonstrate that BA antagonized EC angiogenesis *in vitro*, *ex vivo,* and *in vivo*.

### 3.2. Brevilin A Decreases the Proliferation and Migration of Primary HUVECs

**Figure 2:**
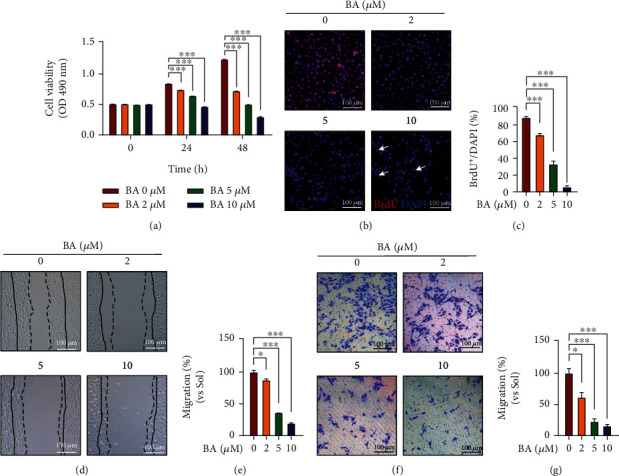
The influences of Brevilin A on proliferation and migration of human endothelial cells. HUVECs were cultured in ECM medium containing 5% FBS supplemented with 10 ng/mL VEGF, and their capabilities of proliferation and motility were subsequently assessed in the presence or absence of BA (0, 2, 5, and 10 *μ*M). (a) MTT assay was used to quantify endothelial cell viability in 24 and 48 h (*n* = 5). (b) Representative images showing BrdU-positive HUVECs with or without BA treatment for 24 h (red: BrdU; blue: DAPI) and (c) quantified BrdU incorporation rate (scale bars: 100 *μ*m; *n* = 3). HUVECs migration was evaluated in (d, e) scratch wound-healing assay (the region between dotted and solid lines on the ipsilateral side indicates the area migrated after 12 h. Scale bars: 100 *μ*m; *n* = 4) and Transwell migration assay (f, g) within an observation interval of 12 h (scale bars: 100 *μ*m; *n* = 3). The graphs summarize the data from at least three times independent experiments. Data are shown as the mean ± SEM. One-way ANOVA with Bonferroni post-hoc test and ^∗^*p* < 0.05, ^∗∗^*p* < 0.01, and ^∗∗∗^*p* < 0.001 versus solvent (Sol).

Angiogenesis is a delicate cell process that requires endothelial cells to undergo active proliferation and migration [38, 39]. The linkages of BA-impaired angiogenesis with EC abilities in proliferation and motility were separately examined. As shown in Figure 2(a), BA significantly inhibited HUVECs proliferation in a concentration-dependent manner within 48 h. Consistently, the dose-dependent reductions of BrdU incorporation were observed in HUVECs with BA treatment for 24 h (Figures 2(b) and 2(c)). Moreover, segmented nuclei were frequently seen in high-dose BA (10 *μ*M) treated cells (arrows indicated in Figure 2(b)), suggesting the potential of BA to influence EC survival.

To avoid interferences from growth inhibition, the impacts of BA on HUVEC motility were determined within a short time interval (12 h). As a result, BA showed dose-dependent inhibitory effects on EC motility were separately verified in a scratch wound-healing assay (Figures 2(d) and 2(e)) and transmigration through a filter (Figures 2(f) and 2(g)). Those data demonstrate that defects in proliferation and migration may functionally contribute to BA-impaired EC angiogenesis.

### 3.3. Brevilin A Induces Apoptosis and Inhibits the PI3K/AKT Signaling Pathway in Primary HUVECs

**Figure 3:**
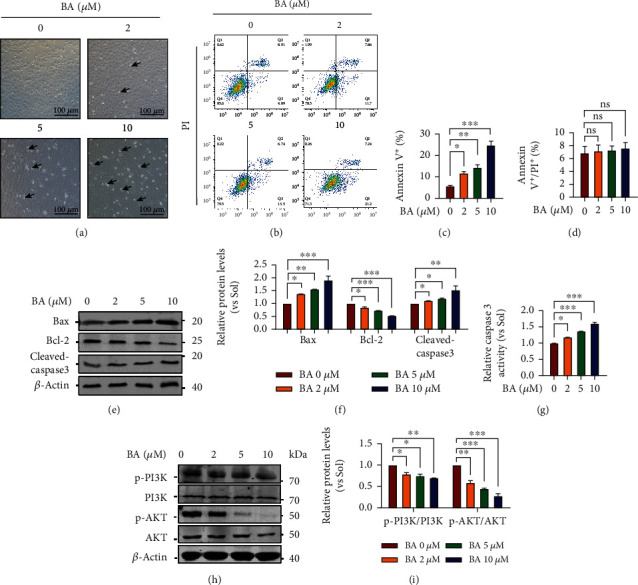
The effects of Brevilin A on apoptosis and PI3K/AKT signaling pathway in human endothelial cells. After the BA application for 12 h, apoptotic phenotypes and signaling pathways were determined in HUVECs cultured in the presence of VEGF (10 ng/mL). (a) Morphological changes of confluent HUVEC cultures observed under a phase-contrast microscope (scale bars: 100 *μ*m; *n* = 5). (b) Flow cytometry evaluated different apoptotic phases via using AV-FITC/PI staining reagents (*n* = 3). (c and d) Bar graphs quantifying the relative distribution of HUVECs in early stage (AV^+^) or late apoptotic stages (AV^+^/PI^+^) (*n* = 3). Western blotting was used to determine (e, f) the protein expression of Bax, Bcl-2, and cleaved caspase-3 (*n* = 3). (g) HUVECs lysates were harvested and further subjected to caspase-3 activity determination (*n* = 5). Western blotting was used to determine (h, i) the protein expression of phosphorylation levels of PI3K and AKT (*n* = 3). The graphs summarize the data from at least three times independent experiments. Data are shown as the mean ± SEM. One-way ANOVA with Bonferroni post hoc test and ^∗^*p* < 0.05, ^∗∗^*p* < 0.01, and ^∗∗∗^*p* < 0.001 versus solvent (Sol).

Given the abnormal nucleus structures observed in BA-treated HUVECs (Figure 2(b)), we thought it might affect EC survival. Indeed, as short as 12 h, BA frequently enables cells to undergo shrinkage as around shape, detachment and floating in monolayer cultured HUVECs (arrows indicated in Figure 3(a)). To further assess the impacts of BA on EC survival status, FACS analysis revealed that BA-enforced HUVECs undergoing early apoptosis as evident by a concentration-dependent increase in AV^+^ cell population (5.9 ± 0.562% in control versus 11.7 ± 0.751% in 2 *μ*M BA, 14.4 ± 1.192% 5 *μ*M BA, and 24.8 ± 1.833% 10 *μ*M BA, shown in Figures 3(b) and 3(c)). While BA seems unable to trigger EC necrosis, a comparable AV^+^/PI^+^ cell population was determined among control and BA-treated HUVECs (Figures 3(b) and 3(d)). To confirm our findings, the activities of caspase-3, an indicator of early apoptosis, were determined. In line with increased active cleavage of caspase-3 (Figures 3(e) and 3(f)), its activity was dose dependently elevated in BA-challenged HUVECs (Figure 3(g)). Consequently, the BA-induced poor survival was molecularly supported by increased expression of Bax and reduced expression of Bcl-2 (Figures 3(e) and 3(f)) as well as inactivation of PI3K/AKT pathway (Figures 3(h) and 3(i)).

### 3.4. Brevilin A Disrupts the Mitochondrial Membrane Potential and Induces ROS Accumulation

**Figure 4:**
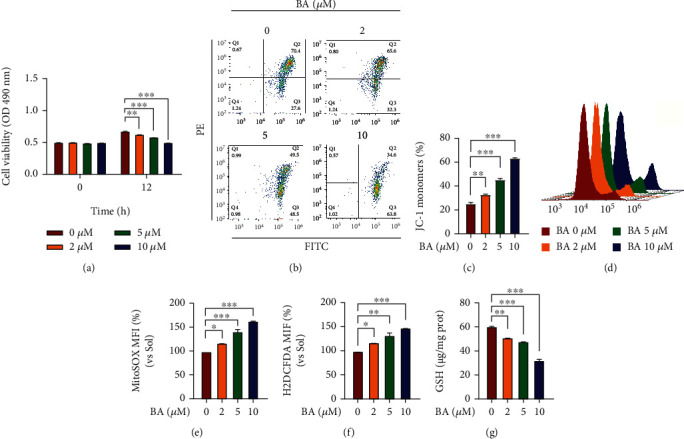
The influences of Brevilin A on redox balance and mitochondrial function in human endothelial cells. After the BA application for 12 h, MMP, MitoSOX, H2DCFDA, and GSH levels were determined in HUVECs cultured in the presence of VEGF (10 ng/mL). (a) Succinate dehydrogenase activities were determined by MTT assay in HUVECs 12 h after BA application (*n* = 5); Meanwhile, flow cytometry was used to assess (b, c) MMP levels by using JC-1 staining (*n* = 3), and (d, e) mitochondrial superoxide contents by MitoSOX probe (*n* = 3). (f) Fold changes of H2DCFDA signals to determine intracellular total ROS levels (*n* = 3). (g) GSH contents in HUVECs (*n* = 3). The graphs summarize the data from at least three times independent experiments. Data are shown as the mean ± SEM. One-way ANOVA with Bonferroni post hoc test and ^∗^*p* < 0.05, ^∗∗^*p* < 0.01, and ^∗∗∗^*p* < 0.001 versus solvent (Sol).

Bax and Bcl-2 are known to localize on the mitochondrial membrane, and dysregulation of its protein expression normally reflects alterations in mitochondrial function. Therefore, the influence of BA on mitochondrial viabilities was evaluated by succinate dehydrogenase activities, and we found that they would be greatly prohibited by BA within 12 h (Figure 4(a)), indicating a resultant damage to mitochondrial function. To verify that, MMP in HUVECs was assessed by JC-1 reagent, and we found that BA would remarkably depolarize MMP as seen by a dose-dependent increase in the JC-1 monomer population (FITC^+^/PE^−^, Figures 4(b) and 4(c)). It is well documented that MMP injury disables the mitochondrial oxidative phosphorylation process, generates excessive ROS, and further trigger apoptosis [40]. In the next step, MitoSOX and H2DCFDA staining results revealed that BA significantly enhanced accumulation of mitochondrial superoxide (Figures 4(d) and 4(e)) and intracellular total ROS (Figure 4(f)) levels in HUVECs, whereas the GSH contents were reversely decreased (Figure 4(g)). Given that the mitochondrial respiratory chain and active NADPH oxidases (NOXs) are the primary sources of ROS in cells [41, 42], we sought to determine whether NOX2 and NOX4 are influenced by BA in ES. Interestingly, the expression of NOX2 and NOX4 did not change in BA-treated groups compared to the control (Figure S2(a)). Taken together, our data suggest BA could disturb intracellular redox balance, especially on mitochondrial superoxide generation, which may account for impaired endothelial functions in survival, growth, and migration and further constrict angiogenesis capacity.

### 3.5. Mito-TEMPOL and N-Acetyl-Cysteine Significantly Reverse Brevilin A-Induced Redox Imbalance, Mitochondrial Dysfunction, and Apoptosis in Primary HUVECs

**Figure 5:**
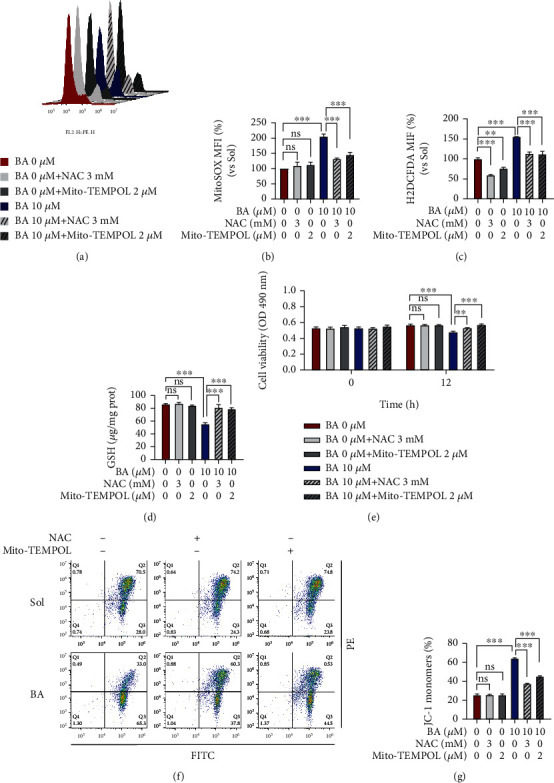
Mito-TEMPOL and N-acetyl-cysteine reverse the consequences of Brevilin A on ROS generation. HUVECs were preincubated with or without Mito-TEMPOL (2 *μ*M) and NAC (3 mM) for 30 min in the presence of VEGF (10 ng/mL), and further administered 12 h treatment with 10 *μ*M BA, and finally subjected to the following analysis. (a, b) Levels of mitochondrial superoxide by MitoSOX probe (*n* = 3). (c) Fold changes of intracellular total ROS and (d) GSH contents in HUVECs (*n* = 3). (e) Succinate dehydrogenase activities were determined by MTT assay (*n* = 4). (f, g) Alternations of MMP levels by using JC-1 staining (*n* = 3). The graphs summarize the data from at least three times independent experiments. Data are shown as the mean ± SEM. One-way ANOVA with Bonferroni post hoc test and ^∗^*p* < 0.05, ^∗∗^*p* < 0.01, and ^∗∗∗^*p* < 0.001 versus solvent (Sol).

**Figure 6:**
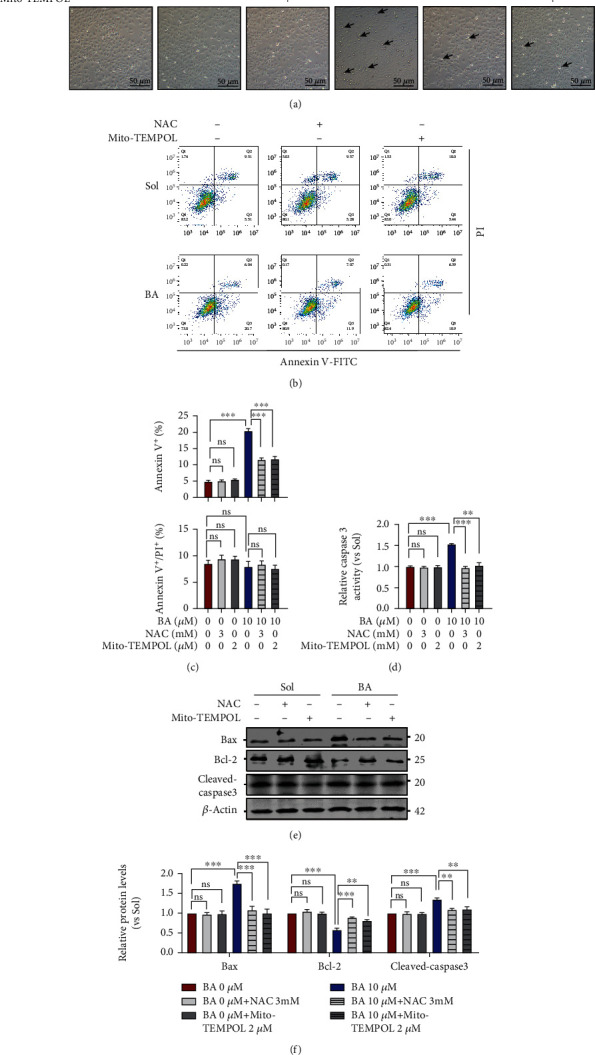
Mito-TEMPOL and N-acetyl-cysteine relieve the consequences of Brevilin A on apoptosis. HUVECs were preincubated with or without Mito-TEMPOL (2 *μ*M) and NAC (3 mM) for 30 min in the presence of VEGF (10 ng/mL), and further administered 12 h treatment with 10 *μ*M BA, and finally subjected to the following analysis. (a) Morphological changes of confluent HUVEC cultures observed under a phase-contrast microscope (*n* = 5). (b) Flow cytometry evaluated different apoptotic phases via using AV-FITC/PI staining reagents (*n* = 3). (c) Bar graphs quantifying the relative distribution of HUVECs in early stage (AV^+^) or late apoptotic stages (AV^+^/PI^+^) (*n* = 3). (d) caspase-3 activation determination (*n* = 3). Western blotting was used to examine (e, f) the protein expression of Bax, Bcl-2, and cleaved caspase-3 (*n* = 3). The graphs summarize the data from at least three times independent experiments. Data are shown as the mean ± SEM. One-way ANOVA with Bonferroni post-hoc test and ^∗^*p* < 0.05, ^∗∗^*p* < 0.01, and ^∗∗∗^*p* < 0.001 versus solvent (Sol).

To address the decisive role of ROS in BA-mediated mitochondrial dysfunctions, Mito-TEMPOL, a mitochondrial specific superoxide scavenger, and N-acetyl-cysteine (NAC), a conventional antioxidant, were employed in BA-treated HUVECs, and functional assays were correspondingly assessed. Firstly, we found that redox imbalance was easily reversed by both Mito-TEMPOL and NAC that occurred in BA-treated HUVECs, as shown by the normalized levels of mitochondrial superoxide (Figures 5(a) and 5(b)), intracellular ROS (Figure 5(c)), and GSH (Figure 5(d)) to those in control cells. Additionally, Mito-TEMPOL and NAC treatment can also significantly reverse BA-induced succinate dehydrogenase inactivation and mitochondrial MMP drop (Figures 5(e)–5(g)). Convincingly, Mito-TEMPOL and NAC were both found to completely rescue BA-induced endothelial apoptotic phenotypes (Figures 6(a)–6(c)), which was further supported by normalized Bax, Bcl-2 and caspase-3 expression (Figures 6(e) and 6(f)), and the caspase-3 activities (Figure 6(d)).

### 3.6. Mito-TEMPOL and N-Acetyl-Cysteine Relieve the Inhibitory Effects of Brevilin A on Endothelial Proliferation, Migration, and Angiogenesis

**Figure 7:**
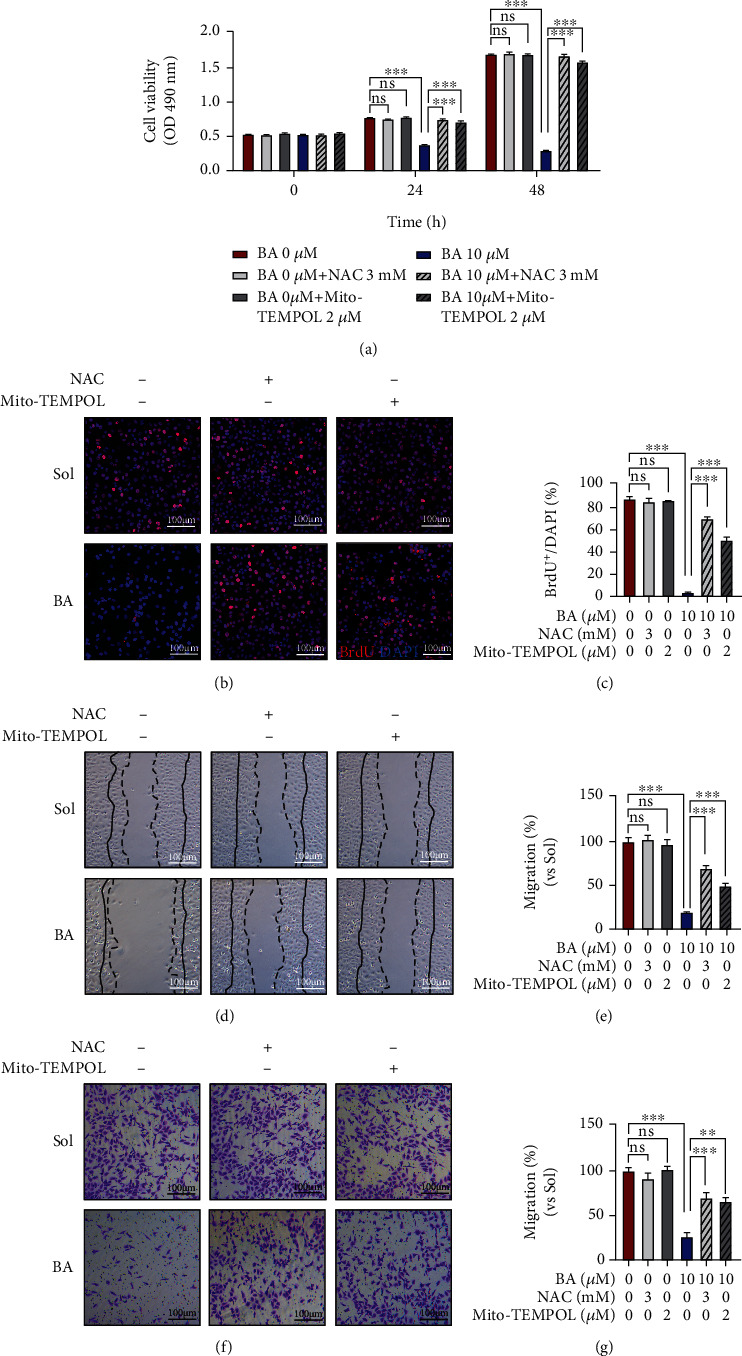
Mito-TEMPOL and N-acetyl-cysteine reverse the consequences of Brevilin A on proliferation and migration. Mito-TEMPOL (2 *μ*M) and NAC (3 mM) were applied into HUVECs 30 min before BA (10 *μ*M) treatment in the presence of VEGF (10 ng/mL) and following cell functions were determined in the indicated times. (a) HUVECs proliferation was determined by MTT assay in 24 and 48 h (*n* = 4). (b) Images showing BrdU incorporation into HUVECs determined in 24 h (red: BrdU; blue: DAPI) and (c) incorporation rate statistics analysis (scale bars: 100 *μ*m; *n* = 3). (d, e) Images of Scratch wound-healing observed within 12 h and quantification of relative migration (the region between dotted and solid lines on the ipsilateral side indicates the area migrated after 12 h (scale bars: 100 *μ*m; *n* = 4). (f, g) Transmigrated HUVECs through a filter were photograph with a time interval of 12 h and the corresponding quantification (scale bars: 100 *μ*m; *n* = 3). The graphs summarize the data from at least three times independent experiments. Data are shown as the mean ± SEM. One-way ANOVA with Bonferroni post-hoc test and ^∗^*p* < 0.05, ^∗∗^*p* < 0.01, and ^∗∗∗^*p* < 0.001 versus solvent (Sol).

**Figure 8:**
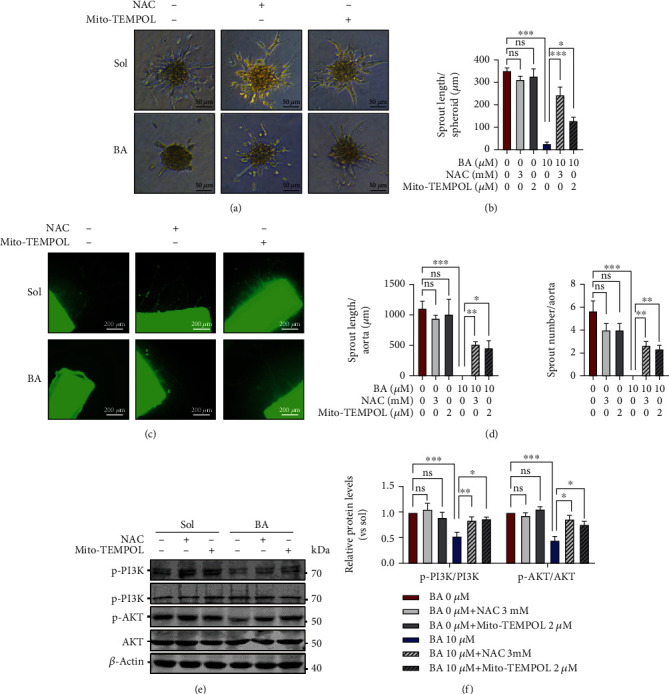
Mito-TEMPOL and N-acetyl-cysteine relieve the consequences of Brevilin A on angiogenesis. (a, b) Representative images showing the endothelial angiogenic abilities were determined by a modified spheroid sprouting assay of HUVECs in the presence of VEGF (30 ng/mL) and accumulated tube length was quantified (scale bars: 50 *μ*m; *n* = 8). (c) The images presenting the outgrowth of native endothelial sprouts from isolated mouse aortic rings and (d) the quantification of sprouts number and total length (scale bars: 200 *μ*m; *n* = 3). (e, f) Western blotting was used to examine the phosphorylation levels of PI3K and AKT (*n* = 3). The graphs summarize the data from at least three times independent experiments. Data are shown as the mean ± SEM. One-way ANOVA with Bonferroni post-hoc test and ^∗^*p* < 0.05, ^∗∗^*p* < 0.01, and ^∗∗∗^*p* < 0.001 versus solvent (Sol).

Next, we further examined the causality of ROS in BA-induced endothelial dysfunction by Mito-TEMPOL and NAC. As expected, Mito-TEMPOL and NAC both exhibited strong abilities to reverse BA-suppressed proliferation (Figure 7(a)) and BrdU incorporation (Figures 7(b) and 7(c)) in HUVECs. Meanwhile, scratch wound-healing assay and Transwell migration assay all demonstrated better recovery of motility in BA-treated HUVECs in the presence of Mito-TEMPOL and NAC (Figures 7(d)–7(g)). Moreover, we found that Mito-TEMPOL and NAC would greatly relieve the inhibition of BA on VEGF-triggered endothelial outgrowth in human EC spheroids (Figures 8(a) and 8(b)) and mice aortic rings (Figures 8(c) and 8(d)). In molecular levels, BA-inactivated PI3K/AKT signaling would also be recovered by Mito-TEMPOL and NAC (Figures 8(e) and 8(f)). Our data demonstrated that irregular mitochondrial ROS overgeneration may primarily account for BA-mediated EC defects in angiogenesis.

**Figure 9:**
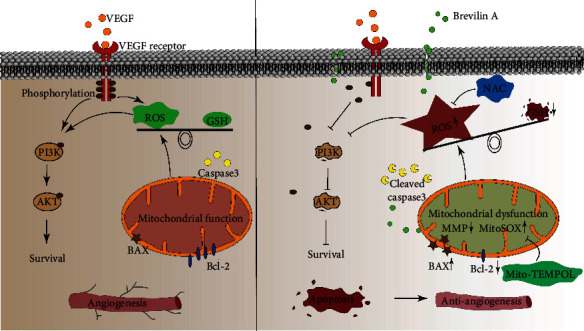
Graphical summary illustrating the inhibitory effects of Brevilin A on angiogenesis.

Therefore, we would like to propose a possible mechanism to explain how BA antagonizes angiogenesis. As shown in Figure 9, VEGF signaling generates proper ROS derived from NOXs and mitochondria, which activates endothelial cell survival, proliferation, migration, and eventually promotes angiogenesis. However, BA would destroy mitochondrial membrane potentials in an unidentified manner, giving rise to overgenerated superoxide in mitochondria. This excessive superoxide would either trigger mitochondria-dependent apoptosis or might propagate throughout cells and disrupt redox balance and impair survival signal transduction (PI3K/AKT). BA-induced excessive ROS contribute to apoptosis, growth arrest, and motility defects observed in endothelial cells and consequently leading to impairment of angiogenesis.

## 4. Discussion

Angiogenesis plays a pivotal role in solid tumorigenesis by promoting the nutrient supply and eliminating metabolic wastes [33]. Carcinoma cells become necrotic or apoptotic cell death in the absence of a circulatory feeding, thus, making it a primary target for the antitumor angiogenic agent [43]. Unraveling the potential therapy of antiangiogenesis led to novel approaches to target tumor. BA, an active component of *Centipeda minima* herbs, has been implicated with desirable antitumor efficacy in suppressing multiple tumor cell motility, survival, and proliferation via inhibiting various signaling pathways and/or inducing ROS-dependent apoptosis in multiple tumor cells [3, 4]. It has shed more light on suggesting BA as a promising antitumor compound. However, its antiangiogenic potential, another important aspect of tumor growth, has been totally unexplored.

In this study, we have shown that BA can efficiently inhibit VEGF-induced tube formation and sprouts outgrowth of primary HUVECs. Furthermore, we found that BA would also effectively abrogate VEGF-stimulated endothelial sprouting from *ex vivo* cultured mouse aortic rings and suppressed neovascularization in VEGF-containing Matrigel plugs implanted in mice. Given that the new vessel formation from the existing vasculature is mainly mediated by VEGF-stimulated stalk cell proliferation and tip cell migration in the first step of angiogenesis [33, 39], the effects of BA on the proliferation and migration in vascular EC were investigated. Not unexpectedly, BA dose dependently repressed VEGF-induced proliferative activity of HUVECs within 48 h. Besides, BA also concentration-dependently declined motilities of HUVECs within 12 h, a shorter observation interval that was designed to be less affected by impaired proliferation. Hence, our data provided solid evidence for the first time to demonstrate that BA is of greatly antiangiogenic ability via inhibiting VEGF-mediated endothelial proliferation and migration.

Neovasculature has previously been reported to be governed by different mechanisms, mainly including the induction of EC apoptosis [44], inhibition of EC remodeling [38], or chemorepulsion of EC [45]. Observation of abnormal nucleus structures and apoptotic-like morphology in HUVECs treated with a high dose of BA (10 *μ*M) reminded us that BA might induce EC death via activating the apoptosis signaling pathway. By using Annexin V/PI staining, FACS analysis confirmed that BA indeed induced an increase in the early apoptosis population without affecting the necrosis cell percentage. The findings could be further supported in molecular levels with BA increased active caspase-3 and Bax expression and inhibited Bcl-2 expression. Consistently, previous studies also demonstrated that enhanced apoptosis is obtained in multiple cancer cells, which contribute to antitumoral actions of BA [3, 4, 6, 7, 10, 12].

Caspase-3 is a well-known executioner of both the intrinsic (mitochondrial-dependent, involving Bcl-2 protein family) and extrinsic (surface receptor-mediated) apoptosis pathways [46]. During the process of mitochondria-mediated apoptosis, the opening of the mitochondrial permeability transition pore reduces MMP, which releases mitochondrial proapoptotic factors into cytoplasm [47]. Proapoptotic factors could then activate caspase cascades, causing nuclear condensation and inducing secondary ROS production, which in turn causes mitochondrial membranal depolarization [40]. Accordingly, our data showed that BA decreased Bcl-2 of antiapoptotic and increased Bax of proapoptotic protein expression, leading to disrupted outer mitochondrial membranal permeability and initiating mitochondrial apoptosis pathway [46]. In line with previous phenotypes in cancer cell lines, we also found that BA significantly induced intracellular ROS production, decreased intracellular GSH, and depolarized MMP in HUVECs [3, 6, 7, 9]. In addition, MitoSOX staining revealed that BA could greatly increase mitochondrial-induced superoxide accumulation in HUVECs, suggesting BA-promoted total ROS contents may be derived, at least in part, from impaired mitochondrial redox balance. To further verify the idea that BA elevated ROS which contributes to mitochondrial-dependent apoptotic pathway, two different anti-ROS reagents were applied. In our present study, NAC (a broad-spectrum antioxidant that directly eliminates ROS, such as hypochlorous acid, hydrogen peroxide, superoxide, and hydroxyl radicals) could mostly remit BA-induced oxidative stresses in intracellular levels of total ROS and mitochondrial superoxide status, which were also associated with recovered GSH contents in HUVECs. Importantly, despite fully inhibiting BA-generated excessive mitochondrial superoxide, Mito-TEMPOL (a specific mitochondrial-targeted antioxidant and superoxide dismutase mimics) was found to almost completely correct BA-induced intracellular redox imbalances as can be seen by a decrease in total contents of ROS and elevation in GSH levels. These findings indicate the importance of mitochondrial ROS in BA-induced apoptosis. Excessive ROS generation is mainly controlled by ectopic-activated NADPH oxidases or release of mitochondrial superoxide [41]. In EC, NADPH oxidases, especially NOX2 and NOX4, which produce superoxide anion that is rapidly converted to hydrogen peroxide, thereby promoting secondary mitochondrial ROS production, which in turn further enhances ROS-dependent VEGFR-2 signaling [48]. As referred from previous studies in tumors revealing that BA would stimulate NOXs expression, we further wondered whether NADPH oxidases involved in BA caused advanced ROS status in EC. However, we found that BA was unable to change the protein expression of NOX2 and NOX4 in HUVECs. This inconsistency may be reasoned by variable genetic backgrounds of cell types used in the individual study; at least HUVECs in our case are normal primary endothelial cell, which are of course greatly distinct from carcinoma cell line such as MCF-7 cells in the previous study [3]. In addition, our data further showed that Mito-TEMPOL and NAC almost reversed BA-induced endothelial apoptosis by normalizing MMP, upregulation of Bcl-2, downregulation of Bax, and consequentially preventing caspase-3 overactivation. Therefore, we propose that BA-induced endothelial cell apoptosis might be largely dependent on the overproduction of mitochondrial superoxide.

Previous studies have demonstrated that BA effectively inhibits the growth of colon adenocarcinoma cell CT26 via promoting autophagy mediated by PI3K/AKT/mTOR signaling [6]. Meanwhile, BA suppresses cell proliferation and induces apoptosis in nasopharyngeal carcinoma cells by suppressing PI3K/AKT/mTOR pathway [12]. BA also remarkably induces oxidative stress in U87 glioblastoma cells by increasing the phosphorylation of stress-activated JNK and p38 proteins [9]. Numerous recent reports have shown that BA effectively inhibits lung cancer, melanoma, and breast cancer cell growth by repressing JAK2/STAT3 pathway [3, 4, 7, 10]. Particularly in homocysteine-mediated apoptosis in EC, an interaction of mitochondrial-dependent apoptotic signaling pathways and PI3K/AKT/eNOS signaling pathways has been established [40]. Thus, we also examined the influences of BA on the expression of those signaling pathways in HUVECs. Notably, we found that BA-suppressed PI3K/AKT pathway, whereas JAK2/STAT3 and JNK/p38 pathways were not affected (data not shown). Expectantly, Mito-TEMPOL and NAC were all readily to recover BA-induced dephosphorylation of AKT and PI3K, consequentially correcting the impaired endothelial behavior in survival, growth, migration, and angiogenesis. Those data suggest the inhibition of PI3K/AKT pathway is secondary to mitochondrial ROS overproduction and may contribute to BA-inhibited endothelial angiogenesis.

Given the fact that our data were obtained in primary HUVECs and wild-type mice or aortic tissues, whether the reproducibility of BA's efficacy and molecular mechanisms in tumoral EC were not included in the current study due to methodological and material limitations, that needed to be addressed in further investigations.

## 5. Conclusion

In conclusion, this study for the first time identified the antiangiogenic effects of BA in vascular EC via inhibiting cell growth, migration, and survival. Mechanistically, BA may directly cause mitochondrial dysfunction, triggering the mitochondrial ROS overproduction, and inhibiting PI3K/AKT pathway and induction of apoptosis. Consequently, our study further suggests that antiangiogenesis serves as a novel insight for BA pharmacological mechanisms on tumor suppression.

## Data Availability

All the data obtained in the current study were available from the corresponding authors on reasonable request.

## Abbreviations

BA: Brevilin A
VEGF: Vascular endothelial growth factor
HUVECs: Human umbilical vein endothelial cells
MMP: Mitochondrial membranal permeability
ROS: Reactive oxygen species
GSH: Glutathione
NAC: N-acetyl-cysteine
EC: Endothelial cells
TCM: Traditional Chinese Medicines
ECGS: Endothelial cell growth supplement.

## Supplementary Materials

Supplementary Materials Figure S1: Brevilin A chemical structure. Figure S2: the effects of Brevilin A on the expression of NOX2 and NOX4.
