# Supplementary material for: Brevilin A Inhibits VEGF-Induced Angiogenesis through ROS-Dependent Mitochondrial Dysfunction
Source: Oxid Med Cell Longev. 2022 Dec 14;2022:5888636. doi: 10.1155/2022/5888636 (PMC9771652; doi:10.1155/2022/5888636)
Supplement: Supplementary Materials — Figure S1: Brevilin A chemical structure. Figure S2: the effects of Brevilin A on the expression of NOX2 and NOX4. [file 5888636.f1.docx]

**Supplementary figures**

**
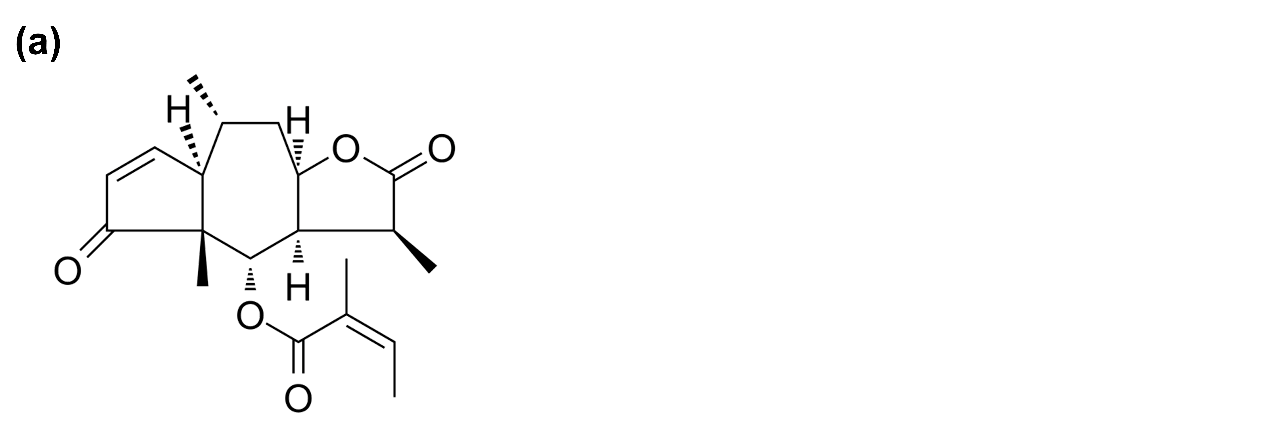
**

**Figure S1. Brevilin A chemical structure.**

**
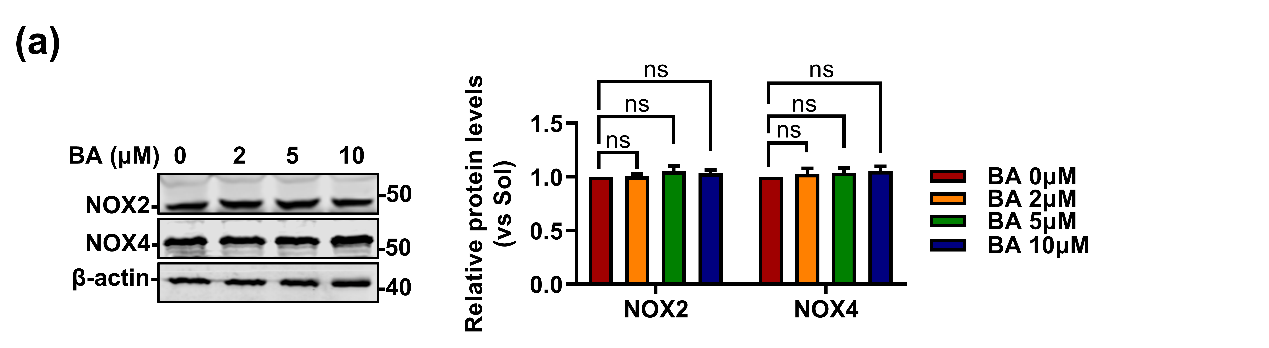
**

**Figure S2. The effects of Brevilin A on the expression of NOX2 and NOX4.** After the BA application for 12 h, the expression of NOX2 and NOX4 was determined in HUVECs cultured in the presence of VEGF (10 ng/mL). (a) Western blotting was used to determine the protein expression of NOX2 and NOX4. The graphs summarize the data from at least three times independent experiments, Data are shown as the mean ± SEM. One-way ANOVA with Bonferroni post hoc test, and **p*<0.05, ***p*<0.01, and ****p*<0.001 versus solvent (Sol).
